# Systematic quantitative characterization of cellular responses induced by multiple signals

**DOI:** 10.1186/1752-0509-5-88

**Published:** 2011-05-30

**Authors:** Ibrahim Al-Shyoukh, Fuqu Yu, Jiaying Feng, Karen Yan, Steven Dubinett, Chih-Ming Ho, Jeff S Shamma, Ren Sun

**Affiliations:** 1 Department of Molecular and Medical Pharmacology, University of California at Los Angeles, Los Angeles, CA 90095, USA; 2 Department of Mechanical and Aerospace Engineering, University of California at Los Angeles, Los Angeles, CA 90095, USA; 3 Department of Medicine, University of California at Los Angeles, Los Angeles, CA 90095, USA; 4 Current Address: Johnson & Johnson Pharmaceutical Research & Development LLC, San Diego, CA 92121, USA; 5 School of Electrical and Computer Engineering, Georgia Institute of Technology, Atlanta, GA 30332, USA

## Abstract

**Background:**

Cells constantly sense many internal and environmental signals and respond through their complex signaling network, leading to particular biological outcomes. However, a systematic characterization and optimization of multi-signal responses remains a pressing challenge to traditional experimental approaches due to the arising complexity associated with the increasing number of signals and their intensities.

**Results:**

We established and validated a data-driven mathematical approach to systematically characterize signal-response relationships. Our results demonstrate how mathematical learning algorithms can enable systematic characterization of multi-signal induced biological activities. The proposed approach enables identification of input combinations that can result in desired biological responses. In retrospect, the results show that, unlike a single drug, a properly chosen combination of drugs can lead to a significant difference in the responses of different cell types, increasing the differential targeting of certain combinations. The successful validation of identified combinations demonstrates the power of this approach. Moreover, the approach enables examining the efficacy of all lower order mixtures of the tested signals. The approach also enables identification of system-level signaling interactions between the applied signals. Many of the signaling interactions identified were consistent with the literature, and other unknown interactions emerged.

**Conclusions:**

This approach can facilitate development of systems biology and optimal drug combination therapies for cancer and other diseases and for understanding key interactions within the cellular network upon treatment with multiple signals.

## Background

Understanding how multiple signals affect cellular functions is necessary in order to be able to understand and control these functions. Extensive studies have been done to address how the activation/inhibition of a particular cellular signaling pathway leads to a specific response. Several challenges limit the ability to study the simultaneous effects of multiple signaling. The complexity and lack of detailed knowledge of cellular systems prevent, in many cases, accounting for the effects of some unknown interactions among pathways or among non-primary signal targets. In addition, genetic or epigenetic alterations between otherwise similar cells can cause a significant difference in their responses. This places additional constraints on the experimental outcomes obtained by analyzing individual components. Furthermore, a critical challenge in the investigation of the effects of multiple signals is the arising complexity associated with the increasing number of signals and their various intensities. Without a systematic approach to replace a large number of time and resource consuming experimental tests, it is difficult to characterize the effects of these signals, to identify appropriate signal combinations.

There has been an increasing interest in examining how various biological activities are regulated by multiple interacting signals [1-4]. Berenbaum introduced a direct search method to optimize cancer chemotherapy regimens [5]. Recently, a method based on stepwise direct search for identifying optimal combination of drugs for pain treatment has been introduced [6]. The method can also be applied in clinical research. More recently, a biased random walk approach called the "modified Gur game" approach was introduced to identify potent drug combinations [7,8]. It is applied towards an objective with a "small" number of experimental trials. While the goal of these studies is to achieve optimization with minimal number of tests, the approach in these studies has several limitations including sensitivity to the design of the automatons driving the random walk and sensitivity to initial conditions. Its capacity to compare the performance of multiple systems will be limited due to the limited amount of obtained information. Moreover, the approach does not guarantee convergence to local or global maxima. In [8], the modified Gur game approach was used to identify a wide range of drug concentrations for which a stochastic search algorithm, differential evolution, was used to maximize an objective function. Although this approach converges to better combinations, the determination of the range of drugs to be used in the combination is sensitive to initial conditions. Another recent and very similar approach uses a deterministic search algorithm for optimization of drug combinations [9]. Determining optimal combinations for systems where a mechanistic model based on mass-action kinetics was recently presented [10]. The use of search algorithms as well as other systems approaches that include the mechanistic mass-action models were reviewed in [11]. Another limitation of these approaches is that they require repetition of the experiment in case the optimization parameters are to be modified or there is a change in the objective function. This limitation becomes significant when considering multi-objective optimization functions in which the objective function is dependent on subjective parameters, resulting in the need to carry over several experiments to determine a suitable set of parameters. Additionally, the convergence of the experimentally applied search algorithms depends on the appropriate selection of the algorithm's parameters. In the absence of a model to enable reasonable selection of the algorithm's parameters, convergence of these algorithms might be compromised. Furthermore, the experimentally applied algorithms can converge to an optima (local or global) that is not very robust to "small" variations in the input signals.

Other recent work on identifying drug combination focuses on identifying mixtures of drugs where the the search space is reduced to use only a single concentration while the search space is increased by searching through a larger number of potential drugs [12,13]. The latter example describes an approach and applies it towards finding promising mixtures for lung cancer.

**Figure 1:**
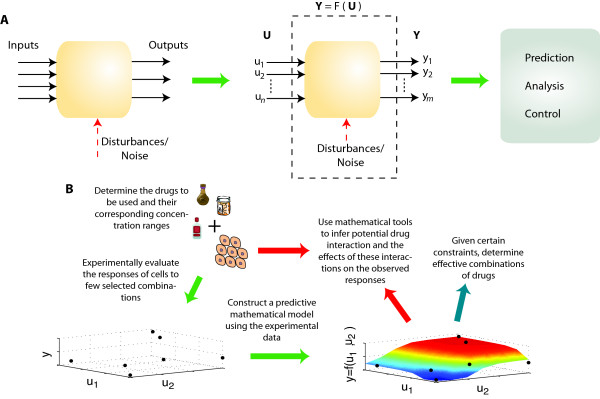
**Summary of approach**. (A) The proposed approach uses input-output data to generate mathematical models capable of predicting the cellular responses to input combinations. The models enable using other mathematical tools for analyzing the cellular responses and for selecting the appropriate combinations of the input signal to drive the system to respond favorably. (B) The desired drugs are combined in certain concentrations and a few combinations are chosen and evaluated experimentally. A predictive mathematical model is generated that can predict the response to all possible combinations. The model can be used to analyze and predict drug interactions and their effects on the observed cellular response and can also be used to determine effective combinations.

In this work, we achieve the desired goals through the integration of data-driven mathematical tools with biological measurements to generate quantitative models of cellular functions (Figure 1). Instead of mapping physical interactions, the resulting model is a quantitative model mapping particular signals to their cellular process responses. The responses represent the net change in certain cellular activities caused by signal interactions within a large and complex network. The model is generated using a suitable mathematical approximation method, which relies on testing a relatively small subset of all possible signal combinations and is capable of predicting the response to the complete set of signal combinations. Through running *in silico *experiments, the model enables analyzing the response of the system to various combinations and determining or selecting subsets of signal combinations that can yield desired cellular responses. The determination of these subsets can be achieved using tools such as stochastic search algorithms and cluster analysis. The proposed approach will facilitate the understanding of fundamental cellular responses, which are system responses reflecting the activity of a complex signaling network controlled by multiple internal and external signals. This approach can promote efficient understanding of cellular functions without intermediates. In addition, the approach allows multiple cell types or other biological systems to be quantitatively characterized, modeled, and compared in parallel. The maximal difference or similarity can be identified using a computational search. It can facilitate the development of drug combination therapies for various types of cancers [14,15].

In comparison to the approaches previously mentioned, the approach introduced in this work overcomes their limitations by identification of the complete response function and carrying out the optimization *in silico*. The cost of carrying such *in silico *experiments is significantly less and is generally faster. The identification of the system response function also provides additional information regarding the potential of using a smaller number of drugs and on key system-level signaling interactions.

## Results

We utilize mathematical tools to characterize the effects of three and four agents on the differential response of cancer and normal cells. The cellular ATP levels of a non-small cell lung cancer cells A549 and primary lung fibroblast culture of AG02603 cells in response to combinations of three chemical agents are measured. Mathematical tools are used to construct predictive models of the cellular ATP levels in response to the combinations. We examine the ability to utilize relatively small numbers of combinations for model generation. The results are extended to study systems of higher complexity with the addition of a fourth chemical agent. The resulting models are used to compare the responses of normal and cancer cell to the same set of combinations. We show that a properly selected combination can result in a significant difference in the respective performance of normal and cancer cells. We also experimentally validate the selected combinations. Furthermore, we show that a combination of chemical agents, if properly chosen, can be more effective than a single agent in inducing a differential response between normal and cancer cells. Using the models, we will examine all the possible lower order mixtures of the four drugs. Moreover, we extract key system-level signaling interactions and compare these interactions between different cell types. We also compare these interactions to known interactions between the drugs.

### Signal-Cellular Response Modeling with a Complete Data Set

**Figure 2:**
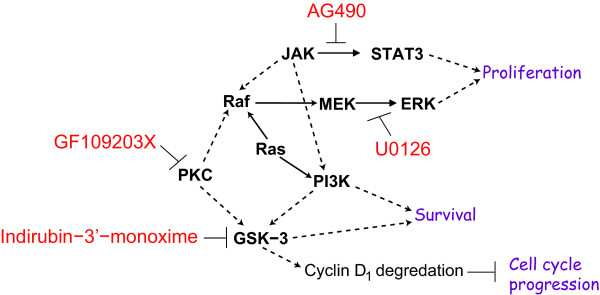
**Simplified pathway and drug interactions**. Shown are simplified pathways targeted in the three and four-drug combination treatment of nonsmall cell lung cancer cells A549 and primary fibroblast AG02603 cells that are already known or reported. The dashed arrows indicate indirect connections.

Inhibition of cell survival and proliferation has been a widely-used approach in cancer treatment [16]. We investigated the combined effect of several drugs that target critical cellular signaling pathways for cell survival and proliferation. Three drugs AG490, U0126, and indirubin-3'-monoxime (I-3-M), which target three distinct while connected signaling pathways critical to most cancer and non-cancer cells, were chosen in our study (Figure 2).

One of the goals of this work is to identify differences in the responses between cancer and non-cancer cells upon the drug treatment. The interactions in Figure 2 are an oversimplified set of interactions of the drugs used. The simplified diagram serves to illustrate some of the known interactions within the cell upon treatment with various drugs. AG490 is a tyrosine kinase inhibitor; U0126 is a MEK inhibitor; and indirubin-3'-monoxime is a cyclin-dependent kinase inhibitor. The drugs are also known to inhibit other targets in addition to the intended target enzymes and as such can lead to unknown interactions. Moreover, each pathway has various interactions with more pathways that are not depicted. The drugs may activate some pathways such as some of the stress responses, directly or indirectly. Thus, the interactions of these drugs and their combinatory effects are difficult to predict.

**Figure 3:**
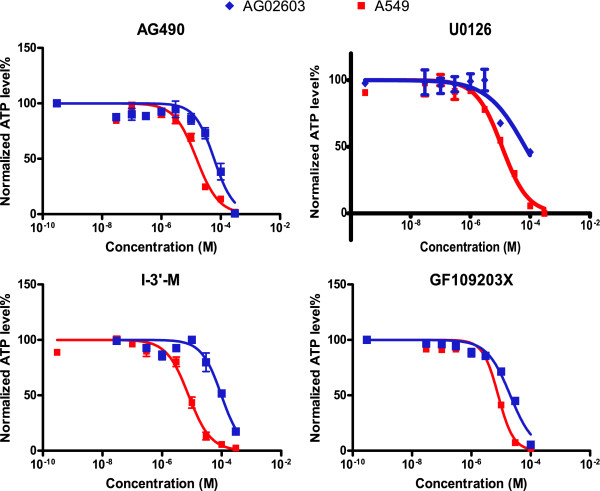
**Single-drug dose response curves**. Shown are the experimental single-drug dose response curves for the four drugs used in the study. The data was used to identify the drug concentrations to be used in combination studies.

Cellular ATP represents one of the most common and essential markers for all live cells. Measuring ATP is a generally accepted quantitative and sensitive assay for assessing the inhibition of cellular growth, proliferation, and induction of cell killing by drugs. The cellular ATP level, which is regulated by multiple cellular pathways, was experimentally quantitated [17]. Total cellular ATP-levels of A549, a non-small cell lung cancer cell line, and AG02603, a normal fibroblast cell culture, were measured 72 hours after drug treatment and normalized to untreated cellular ATP-levels. The drug response dose curves were measured for each of the three drugs (Figure 3). The three drugs (each with eight different concentrations including zero) comprised 512 possible combinations in total. The doses of individual drugs were chosen based on the individual dose-response curves and covered the concentration ranges that resulted in minimal to maximal cell inhibitory effect (Table 1).

**Table 1:** Concentrations of the drugs used in the three and four drug treatments.

A	Drug Name	Drug Concentration
AG490		0, 0.3, 1, 3, 10, 30, 100, 300 (uM)
U0126		0, 0.1, 0.3, 1, 3, 10, 30, 100 (uM)
Indirubin-3'-monoxime		0, 0.3, 1, 3, 10, 30, 100, 300 (uM)

**B**	**Drug Name**	**Drug Concentration**

AG490		0, 1, 3, 10, 30, 100, 300 (uM)
U0126		0, 0.1, 0.3, 1, 3, 10, 30 (uM)
Indirubin-3'-monoxime		0, 1, 3, 10, 30, 100, 300 (uM)
GF109203X		0, 0.3, 1, 3, 10, 30, 100 (uM)

(A) Shown are the eight concentrations (including zero) of each of the three drugs (AG490, U0126, Indirubin-3'-monoxime) used in the three-drug treatments of A549 and AG02603 cells. (B) Shown are the seven concentrations (including zero) of each of all four drugs (AG490, U0126, Indirubin-3'-monoxime, GF109203X) used in the four-drug treatments of A549 and AG02603 cells.

The ATP level in response to all of the 512 drug combinations was experimentally measured in lung cancer A549 cells and in primary lung fibroblast AG02603 cells. The fibroblast cells were derived from normal healthy tissue and are not cancer cells. There are several mathematical methods that can be used to generate models of input-output data. Here we provide a comparison of some of these methods in view of the function approximation problem considered. The methods include two neural network structures and two linear regression models. The neural network structures are a single layer multi-layer perceptron (MLP) and a cascaded neural network [18,19]. We have examined different numbers of neurons per layer for each of these neural network structures. The results below show that a four-neuron single-layer MLP is sufficient for the purposes of this work. For the cascaded network, two layers with a single neuron per layer were sufficient. Networks with more neurons per layer also produced satisfactory results. The two linear regression models involve different nonlinear regressors. The first one uses interaction terms that are pairwise and k-wise products of all the concentrations of the drugs. The second is a quadratic response surface that uses only pairwise products and quadratic terms of the concentrations (See the Methods Sections).

**Figure 4:**
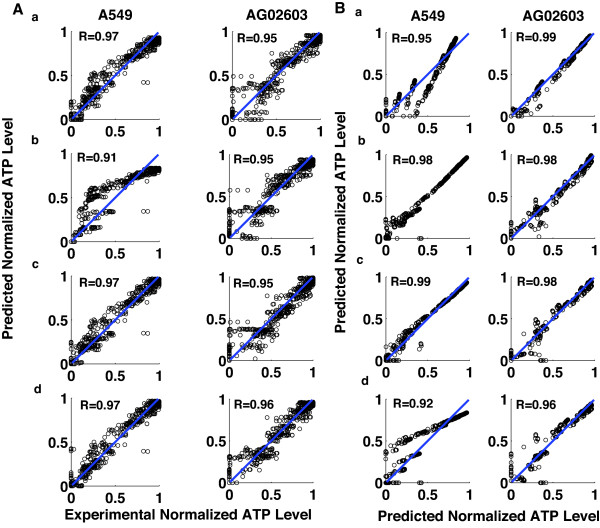
**Three-drug model fitting**. The figure shows an evaluation of modeling the responses of A549 and AG02603 to combinations of three drugs. (A) The panels show a plot of the model predicted cellular ATP levels versus the experimentally measured values. Cellular ATP-level predictive models for A549 and AG02603 cells were developed using a number of different methods. a. A linear regression model that uses pairwise products of concentrations and quadratic terms (QRF). b. A linear regression model with n-wise products of concentrations (LR). c. A cascaded neural network with two single-neuron layers (Cascaded NNet). d. A four-neuron single layer multi-layer perceptron artificial neural network (MLP). The models are based on fitting 80 out of 512 combinations and the figure shows the predicted versus experimental values for all 512 combinations. The correlation between the experimentally tested cellular ATP-level (x-axis) and the predicted cellular ATP-level (y-axis) is shown. The circles in the graphs represent individual data points. The diagonal line represents a perfect fit between the experimental and predicted data. (B) Comparison between the predicted normalized ATP levels of different models. The predicted ATP levels for different models are plotted against each other. a. QRF versus LR. b. Cascaded NNet versus MLP. c. QRF versus MLP. d. LR versus MLP. The correlation coefficients between the different methods are shown.

The different models were trained against 80 out of 512 points with the goal of minimizing the mean square error of prediction. The outputs of the models are processed through a saturation function to limit outputs to the interval [0,1]. The trained models can predict the responses to all 512 combinations with high fidelity. The correlation coefficients between the predicted normalized ATP levels and their corresponding experimentally measured values are higher than R = 0.91 (Figure 4A). Looking at only the points that were not used for training, i.e., the 432 points, the correlation coefficients between the predicted normalized ATP levels and their corresponding experimentally measured values were also high (Table 2). We have also examined how the different models compare to each other by measuring the correlation between their predicted values (Figure 4B). Examining the correlation between the different models, we see that there is also strong correlation with R-values higher than 0.94.

**Table 2:** Correlation coefficients between the 432 points not used for training and the corresponding experimentally measured values.

Cell Type	QRF	LR	Cascaded NNet	MLP
AG02603	R = 0.96	R = 0.95	R = 0.95	R = 0.96
A549	R = 0.96	R = 0.92	R = 0.97	R = 0.98

**Figure 5:**
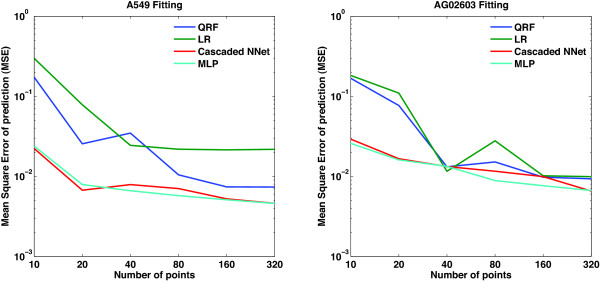
**Model accuracy as a function of modeling method and data size**. The figure shows the effects of using different sizes of data sets for fitting the models and the effects on the model accuracy as measured by the mean square error of prediction. The mean square error of predicted data using four different methods and using 10, 20, 40, 80, 160, and 320 data points, for both A549 and AG02603 cells are displayed. The four methods are two linear regression methods and two neural network methods. The linear regression methods include one that uses pairwise products of concentrations and quadratic terms of the concentrations (QRF), and the other uses the n-wise products of concentrations of drugs as interaction terms (LR). The two neural network methods are a cascaded neural network with two single-neuron layers, and a four-neuron single-layer multi-layer perceptron (MLP). As the number of points used to generate the model increases, the mean square error decreases.

Experimental testing of all possible combinations can be a costly process. Whenever the response of the biological system is smooth enough, we can utilize a smaller number of combinations to map the entire response surface. In this regard, we have examined the effects of using varying numbers of points to fit the models on the accuracy of prediction. The four methods discussed above were considered and models using 10, 20, 40, 80, 160, and 320 points were fitted. To mimic an actual experimental setup, the points were randomly selected out of the 512 possible combinations using a uniform distribution. The mean square error of prediction for each of the methods and fitting data for both cell types indicate that increasing the number of points reduces the mean square error (Figure 5).

However, no significant improvement in the errors are observed for models with more than 80 points. Using a small number of points results in poor prediction with the linear regression models, the interaction model and the quadratic models. In the absence of post-processing of the data by passing through a saturation function, the mean square error of prediction of the linear regression models become significantly worse. Between the two linear regression models, the quadratic model performs better than the interaction model, potentially due to the added quadratic terms, suggesting the nonlinearity of the response of these cells to the drug combinations used. These models also have higher mean square errors than the neural network models. The differences between the mean square error of these models tend to diminish as the number of points used increases. The two neural network models were comparable overall.

The number of data points required to generate a valid model relies on factors including intrinsic signal-response relationships for individual cell cultures and the experimental measurement error. In addition, the smoothness of many signal-response relationships enables the modeling to rely on less dense mapping over small ranges of signal concentrations. Our results suggest that with a proper mathematical modeling method, the effect of signal combinations can be systematically described through randomly testing a relatively small percentage of signal combinations within specified concentration ranges.

### Characterization of Signal-Cellular Response Relationships in Systems of Higher Complexity

**Figure 6:**
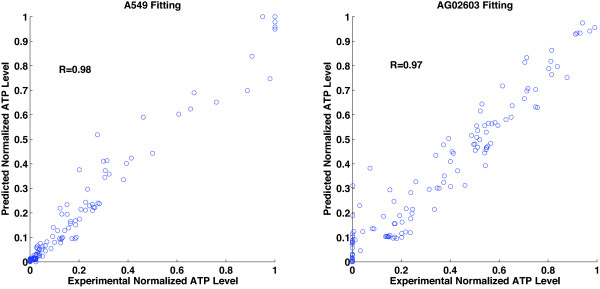
**Four-drug model fitting**. The figure shows the model-predicted ATP-levels versus the experimentally obtained values for four drugs combinations. The model used is a four neuron single layer neural network model that was fitting using a data set of size 148 out of 2401 combinations. The correlation between the experimentally tested cellular ATP-level (x-axis) and the predicted cellular ATP-level (y-axis) is shown. The circles in the graphs represent individual data points. The diagonal line represents a perfect fit between the experimental and predicted data.

**Figure 7:**
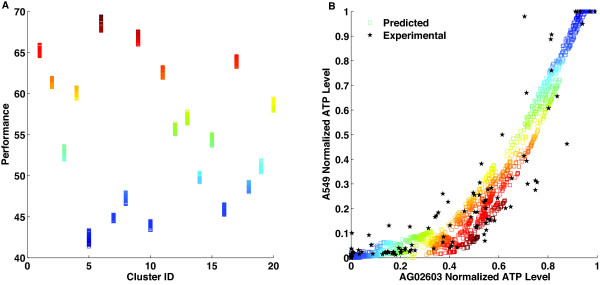
**Analysis of drug combination differential responses**. The figure presents the analysis results to identify combinations of drugs that can maximize a performance function reflecting the desired objective of minimizing the killing of AG02603 cells and maximizing the killing of A549 cels (see methods section for the details on the performance functions. (A) Results of k-means clustering analysis of the performance of four-drug combinations. Points are grouped together to minimize the distance to the mean of that group. Each group is given a identification number (ID). The x-axis shows cluster IDs; the y-axis shows the performance score of each drug combination. The heat map is a function of the performance for each point. (B) Cell ATP-level (x-axis: AG02603; y-axis: A549) upon four-drug combination treatments. The red stars represent the experimentally tested 148 drug combinations. The squares represent the whole set of 2401 possible drug combinations with the heat map colors reflecting the performance of each combination.

The simulated models capable of systematically describing the signal-cellular response relationships for various cells enable the comparison of the cell-type specific differences in cellular responses to multi-drug treatments. An interesting question is whether our approach can simulate more complex systems with a relatively small set of experimental data, and whether efficient multi-drug combinations that lead to a high level of A549 cell inhibition while preserving AG02603 cells can be identified among more drugs. In this regard, we added GF 109203X, a PKC inhibitor, to the three drugs tested in the above section. The addition of a fourth drug increases the search space to 2401 combinations (four drugs with seven concentrations each). Models representing the relationship between the four drugs and the ATP-levels of A549 and AG02603 cells were generated based on 148 experimentally tested combinations. We fitted a single-layer neural network with four neurons using the experimental data. The correlation coefficients between the predicted data from this model and the experimental data were 0.98 for A549 and 0.97 for AG02603 (Figure 6). Overall, there is a very high agreement between the predicted and experimental results (Figure 7).

We have also investigated the ability of these models to predict the experimental data obtained in the three drug combinations experiment. These combinations correspond to four-drug combinations where the concentration of the fourth drug is set to zero.

Correlation coefficients between the 512 data points and their predicted values were 0.86 for A549 cells and 0.88 for AG02603. These correlation coefficients are quite reasonable given that the two experiments were conducted several months apart and the model was trained on a separate and independent data set. This provides evidence of the ability of our models to predict cellular responses despite the relatively small number of data points used to generate these models.

### Effects of Single Signal Versus Multiple Signals

Although all four drugs inhibit cellular functions common in both cell types, combinations of these drugs may result in a significant difference in cellular ATP-levels between A549 and AG02603. Inhibition of A549 cells and preservation of AG02603 cells using the same drug combination constitute two conflicting objectives. Our goal of identifying the drug combinations that effectively satisfy the above objectives can be realized by utilizing a multi-objective search or optimization technique. A performance function combining the relative importance of each of the two conflicting objectives is introduced (Materials and Methods). The drug combinations resulting in the highest performance are considered the best drug combinations of a given set of drugs with corresponding concentrations.

Identification of the best performing subset is achievable through various methods. Enumeration and sorting of all possible combinations and their corresponding performances is one method. Alternatively, we can use a clustering algorithm such as a k-means clustering algorithm to group combinations with similar performances [20,21].

**Figure 8:**
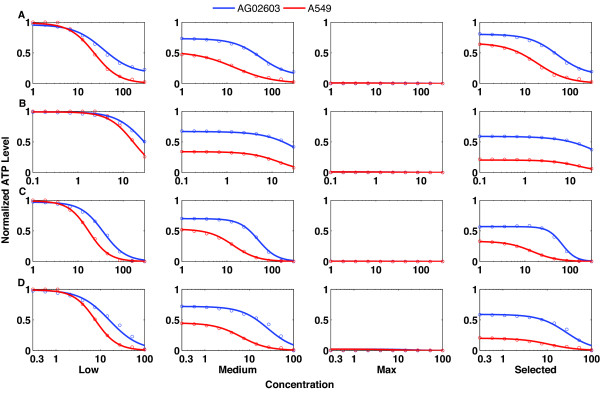
**Single-drug response curves**. The figure shows an evaluation of the model-predicted ATP-levels of A549 and AG02603 when the concentrations of three drugs are held constant while the concentration of the fourth drug is varied. The values of the concentration at which the three drugs are held constant are shown in Table 3. Shown are the single-drug effects of increasing concentrations of (A) AG490, (B) U0126, (C) indirubin-3'-monoxime and (D) GF 109203X on cellular ATP-level with respect to different levels of the other three drugs in the treatment. Here the second row fourth column subfigure corresponds to the cellular ATP-levels of A549 and AG02603 to varying concentrations of U0126 while the remaining three other drugs are held at the selected concentration in Table 3.

Clustering the points into 20 different groups (Figure 7A), we find that the points with the highest performance are associated with low A549 ATP-level and moderate to high AG02603 cellular ATP-level (Figure 7B). The heat map on both panels is a function of performance and the best performing combinations are highlighted in dark red (Figure 7). The effect of drug combinations on the difference in the ATP-level of A549 and AG02603 cells can be clearly seen in Figure 8 which shows the predicted individual dose-response curves of both cell types in the presence of different levels of the other three drugs. The levels chosen are zero (low) concentrations, medium concentrations, maximum concentrations, and a selected combination from the subset of combinations identified to maximize the introduced performance function (Table 3). A significant difference in the response was observed at the selected combinations. This result illustrates how a properly chosen combination can result in a response unachievable individually by any of the drugs. As evident in Figure 8, the response to any individual drug on its own was small. However, a properly selected combination of the same four drugs yields a significant increase in the response.

**Table 3:** Shown are the concentrations used for for generating the drug response curves.

Drug Name	Low	Medium	Maximum	Selected
AG490	0	10	300	30
U0126	0	1	30	0.3
Indirubin-3'-monoxime	0	10	300	10
GF109203X	0	3	100	0.3

**Figure 9:**
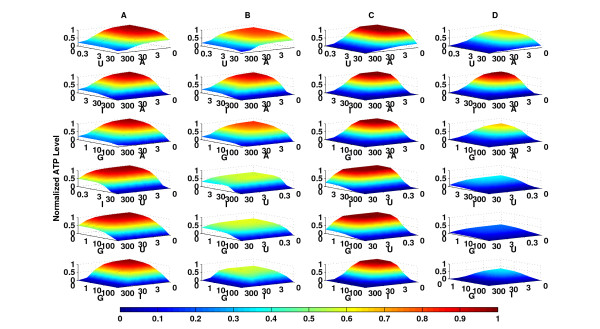
**Two-drug interactions**. The figure shows an evaluation of the model-predicted ATP-levels of A549 and AG02603 when the concentrations of two drugs are held constant while the concentration of the other two drugs are varied. The first and third columns (A) and (C) correspond to ATP levels of AG02603 in response to variations in different pairs of drug. The concentrations of the other two drugs are fixed at zero for column (A) and a selected combination (Table 3) for column (C). The second and fourth columns (B) and (D) correspond to ATP levels of A549 in response to variations in different pairs of drug (rows). The concentrations of the other two drugs are fixed at zero for column (B) and a selected combination (Table 3) for column (D). The figure shows that at the selected combination, the difference between the response surface of AG02603 and A549 is significantly higher than when the difference at zero concentrations.

Additionally, we investigated the effects of pairwise combinations of drugs on the responses of both A549 and AG02603 cells. The remaining two drugs were fixed at one of two cases, zero or a selected optimal concentration (Table 3) from the set of combinations identified to maximize the performance functions. Similar to single drug responses, there is a significant difference between combinations of two drugs and combinations of 4 drugs (Figure 9). The data illustrates that there is a significant difference between normal and cancer cell ATP levels when two drugs are used with the other two fixed at a selected combination concentration versus zero concentration. In addition, the data shows that using a four drug combination increases the effective range or therapeutic windows of the two drugs when compared to two drug combinations.

### Examining the Efficacy of Mixtures of Two, Three, and Four drugs

**Figure 10:**
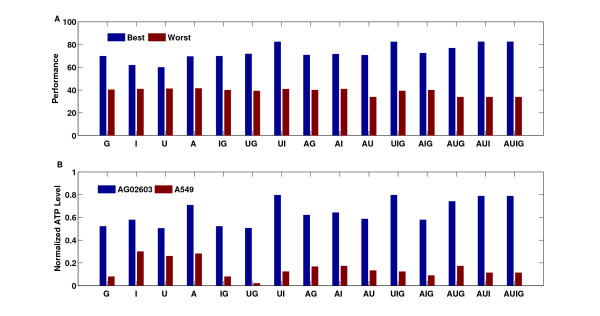
**Evaluation of the best combinations of all lower order mixtures of the drugs**. (A) The performance of the best combination for single drugs as well as combinations of two, three, four drugs are shown. Also shown are the performances of the worst possible combinations. (B) The normalized ATP level for the best combination for all single, two, three, and four-drug mixtures are shown.

The availability of the model enables examining all lower order mixtures of drugs and their potential performances. Testing four or more drugs at time can enable efficient identification of effective lower order mixtures of the drugs. Based on the model, we evaluated the performances of all possible mixtures of drugs at varying concentrations. We identified the best achievable performance for each mixture (Figure 10). The results show that there is an improvement in the achievable performance as more drugs are used.

However, this improvement becomes less as four drugs are used instead of three. The results also show that the improvement is more significant for certain mixtures of drugs, e.g., the performance of the mixture of AG490 and I-3-M is better than the performance of AG490 and U0126. A similar observation can be made about three-drug mixtures where the mixture of AG490, I-3-M and GF109203X performs better than the three other three-drug mixtures. This information is not known a priori and without this approach, determining the best mixtures requires testing of each mixture independently. This can be a lengthy and costly process. Moreover, if one elects to randomly select combinations for the mixtures then there is no guarantee that the combination or mixture can have a good performance. In fact the random combination and mixture can perform quite poorly (Figure 10A). Hence, an effective approach for identifying mixtures of drugs and effective combinations can be very useful in this regard.

The potency of our approach can be illustrated by examining the performance of this approach versus more simple approaches. An example of such approaches is combining the drugs at the best single-drug concentrations. The best concentrations for each single drug were 30uM, 30uM, 30uM, and 100uM for AG490, U0126, I-3-M, and GF109203X respectively. Combining the drugs at the best single-drug concentrations results in a poor performance of 44.48 corresponding to zero normalized ATP levels of A549 and AG02603. One can argue that this combination can be very toxic because of the combined high concentration of each of the drugs. However, there is no simpler way to reduce the concentration of some or all drugs to achieve a better performance. Additionally, an argument can be made for the selection of the mixture of two drugs by picking the two best single drugs. In our case, this would correspond to mixing U0126 and I-3-M. This mixture does not perform as well as the mixture of AG490 and I-3-M (Figure 10). Again, there is no simpler approach for the selection of such mixtures. The approach we introduced attempts to answer to this need and provides a systematic approach that can be used to identify the best combinations for mixtures of two, three, and four drugs. In a more general setting, the approach enables identification of efficient combinations and mixtures for any number of *n*-drugs.

Validation of selected top performing combinations (30 uM of AG490, 0.3 uM of U0126, 10 uM of I-3-M, and 0.3 uM of GF109203X), (30 uM of AG490, 0.3 uM of U0126, 30 uM of I-3-M, and 1 uM of GF109203X), and (30 uM of AG490, 1 uM of U0126, 10 uM of I-3-M, and 0.3 uM of GF109203X) showed that the experimental ATP levels of A549 and AG02603 were 0.2 and 0.65, 0.13 and 0.51, and 0.13 and 0.5 respectively. This is in agreement with the predicted values for A549 and AG02603 of 0.21 and 0.59, 0.08 and 0.51, and 0.2 and 0.58 respectively.

### Drug-Drug Interactions Affect the Observed System Responses

Modeling of multi-signal induced cellular responses can also be used to study key system-level signaling interactions between the applied signals and their effects on the system outputs. To that end, consider the linear regression model with interaction terms. This model uses a total of 15 regressors that are the concentrations of the drugs, six pairwise interaction terms, four three-drug interaction terms, and one four-drug interaction term. This is a large number of regressors and, potentially, only a portion of these regressors are necessary to describe the variation in the data. Reduction of the regressors matrix *X *can be achieved using principal components analysis. Partial least squares can also be used and in this case the reduction in the regressors matrix takes into account variation in the output. However, the components obtained with principal component analysis or partial least squares models would be hard to interpret given the large number of regressors. Instead, we pursue a subset selection algorithm based on all the possible subset regressions [22]. The algorithm provides the best models of 1, 2, 3, . . ., 15 regressors. In total, the algorithm provides the best 15 models out of 2^15 ^- 1 possible models.

**Figure 11:**
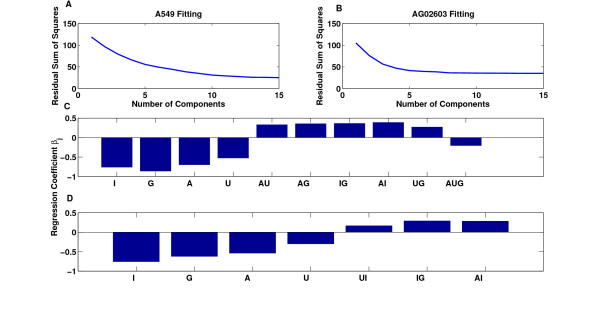
**Analysis of models with varying numbers of regressors**. Shown are the results of best subset regression algorithm which evaluates the best models of 1, 2, . . ., *n *regressors. (A) The residual sum of squares of the best models of A549 data as a function of the number of components (regressors). There is no significant decrease in the residual sum of squares for models with more than 10 regressors. (B) The residual sum of squares of the best models of AG02603 data as a function of the number of components (regressors). There is no significant decrease in the residual sum of squares for models with more than 7 regressors. (C) The regression coefficients for the best 10 component model of A549 data. The components involve single drug concentrations, pairwise and three-drug interactions. (D) The regression coefficients for the best 7 component model of AG02603 data. The components involve single drug concentrations and pairwise interactions.

**Figure 12:**
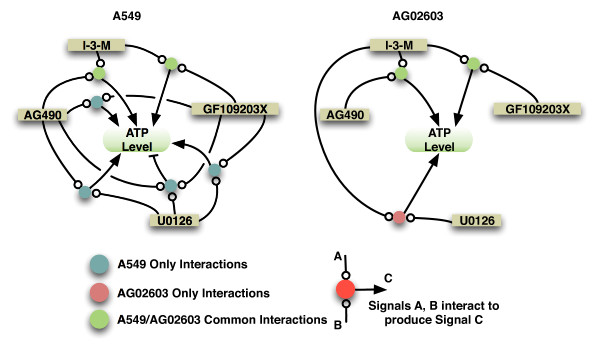
**System-level signaling interactions in A549 and AG02603 cells**. Shown are some model-predicted system-level signaling interactions upon treatment with multiple drugs. The figure shows the interactions that are specific to A549, the interactions that are specific to AG02603, and the interactions common to both A549 and AG02603 cells. Direct arrows between the drugs and the ATP levels exist and are omitted to simplify the diagram.

The residual sum of squares of the best models shows that there is no significant reduction in the residual sum of squares for models with more than 10 regressors for the A549 data, and for models with more than 7 regressors for the AG02603 data (Figure 11A,B). The regressors used in these models correspond to single-drug concentrations, pairwise interactions, and three drug interactions for the the A549 data. Whereas the regressors for the AG02603 data included only single-drug concentrations and pairwise interactions (Figure 11C,D). The effects of these interactions vary between positive and negative. The individual concentrations and three-drug interactions have a negative influence, and the pairwise interactions have a positive influence on ATP levels. Examining the interaction terms, these interaction terms can be grouped into three categories, interactions that only occur in A549 cells, interactions that only occur in AG02603 cells, and interactions that are common to both cell types (Figure 12).

## Discussion and Conclusions

We demonstrated the integration of an efficient mathematical approach for a systematic quantitative characterization of the effect of multi-signal combinations in two different cell types. Our method enables the establishment of accurate models to directly connect multi-signal combinations and their effects through a learning process. Only a small percentage of total data points are required to be experimentally tested to establish a predictive model that is capable of simulating the effect of all possible signal combinations.

The resulting predictive model is also able to systematically reveal the inter-drug interactions which are often non-linear relationships. Such a model is necessary for multi-drug combination optimization as the optimal combination, upon the addition of a new drug, cannot be achieved simply by testing various amounts of the new drug added to the previously optimized combination. Moreover, the approach allows for examining all lower order mixtures of the drugs and for evaluating the effectiveness without added experimental overhead. This enables efficient selection of drug combinations of different drugs particularly as the interactions between the different drugs vary. The approach enables systematic selection of input signals (drug combinations) that can achieve desired therapeutic goals. Experimental validation of selected top performing combinations presents additional evidence of the validity and utility of the approach introduced.

Our approach requires a relatively small number of experimental measurements. With the development of large scale-high throughput measurement systems, our approach will be necessary and more efficient, e.g., when the number of inputs increases. Additionally, it will allow for integration into automated machines for testing and analysis of various biological systems. The addition of other cellular outputs can be another factor in favor of using this approach.

### Mathematical Modeling Methods

We demonstrated the utility of various mathematical modeling approaches for the purposes of modeling and predicting multi-signal induced cellular responses. Linear regression with models including interaction and quadratic terms were capable of producing powerful predictive models. The usefulness of linear regression methods and subset selection algorithms was also demonstrated as they enabled determination of key system-level signal interactions that result in the observed cellular responses. Alternately, neural network models performed generally better for fitting the data particularly when fewer data points were available to fit the models. Additionally, different structures of neural networks were almost equally powerful in producing the models. This present ample opportunity to exploit different structure of networks to mimic more realistic interactions within the cell given such a priori data. The choice of the best method for fitting the model is dependent on many factors. While in some cases simple linear regression can be used, in other cases nonlinear regression methods are necessary to get satisfactory models. Neural networks represent powerful computational models with great flexibility. Different types and configuration renders them useful for a wide range of problems including dynamic prediction using, e.g., recurrent neural networks. This presents an additional use for neural network to build on a wide range of applications in biological and medical research [23-34].

It is important to note here that the novelty in this work is not this particular use of artificial neural networks. Rather, it is the use of a system-level model-based approach to study the effects of a large number of signals on various cellular processes and to use that as a basis for selection of the retrospective optimal input signals. Our method resembles a general systems biology approach that can be utilized to address a broad range of biological questions. The method enables comparison of multiple system performances through modeling of their responses. The advantage of our method becomes critical when input signal combinations are characterized for the development of effective *in vivo *therapies, in which case the limited experimental scale imposes restrictions on the number of possible drug combinations to test. By largely reducing the number of tests required, our approach can greatly facilitate the development of clinically applicable treatments.

For the drugs and cell types chosen, our results showed that four drugs did not provide a significant improvement over three drugs. However, this is not a general result and the causes of this observation are not well known and pose an interesting problem to be examined in a separate study. A potential cause is that the signaling pathways affected by some of the drugs are saturated potentially due to sharing of a common target between two of the drugs. In other studies we are working on with different drugs and cells, four drugs result in an improvement over three drugs and five drugs also improve on four drugs.

### Mechanistic Reasoning

Our modeling approach enabled identification of key system-level signaling interactions that contribute to the observed responses. These interactions highlighted potential differences in the signaling networks of different cell types as demonstrated. While some signaling interactions were common to the two cell types studied, there were other interactions that were specific to each cell type. Many of these interactions were known a priori. For example, the interactions between U0126 and GF109203X, I-3-M and GF109203X, AG490 and I-3-M, as well as AG490 and U0126 and GF109203X. There are other interactions that were not known a priori such as the interaction between U0126 and I-3-M. Thus the prediction of such interactions can guide experimental identification of the molecules mediating the interactions.

In the work presented, we used mathematical tools to construct a predictive model of cellular outcomes. The method was developed in experimental systems that only involved single output measurements. However, the method is a general method that can be used to construct models of the effects of multiple signals on various cellular outcomes, including signaling intermediates. Molecules from various cellular pathways, "intermediates", can serve as candidates for measurement and quantification [1,35,36]. Moreover, measuring these cellular outcomes and intermediates at various time points enables the construction of predictive dynamic models. The incorporation of time provides the model with predictive capability similar to those of ordinary differential equation models, though without a priori assumptions or knowledge about the molecular interactions. The introduction of more cellular outcomes presents the opportunity to utilize additional tools that can infer sets of rules which can provide, at least in part, descriptive reasoning of some of the internal interactions within the cell [37-39].

### Implications on the Design of Combination Therapies

Combinational therapies have attracted significant research efforts. Combination therapy for cancer is one example. The challenges associated with cancer treatment range from a lack of understanding of fundamental pathogenic mechanisms to practical experimentation limitations. Cancer is usually caused by multiple mutations and alterations of multiple signaling pathways which pose an extra challenge when defining the mechanisms underlining cancer development [40]. In addition, there is extensive heterogeneity of tumors among individual patients. Thus there are multiple potential targets for cancer therapy, resulting in an increasing need for distinct therapeutic agents and their combinations. Furthermore, drug resistance, arising from a subpopulation of original tumor cells or from subsequent mutations under the selection pressure of drug treatment, has been a major hurdle in cancer treatment. Drug combination therapy is emerging as a potentially effective approach to prevent drug resistance, as well as achieve higher efficacy and lower toxicity [41-43].

However, the design of combination therapies based on studies of the response to individual drugs might not lead to the desired outcomes as interactions between the various drugs can lead to unknown outcomes. The emerging toxicities can be a major hurdle to the development of combination therapies. Therefore, it is important to consider system level signals or outputs that are representative of the state of the cell in addition to cellular intermediates targeted by the drugs. As such, it is important to investigate whether and how a combination of drugs can lead to a better system response compared to any of the individual single-drug responses.

Development of drug combination therapies for cancer can lead to more effective therapies to overcome drug resistance and achieve maximal drug efficacy. The demanding task is to find a combination that is maximally effective on cancer cells with minimal effect on non-cancer cells. The method introduced in this work provides the ability to study the system-level effects of combinations of drugs and use the data to select combinations that can lead to desired outcomes by comparing the mathematical models of multiple cellular types. Furthermore, the method is capable of studying and characterizing experimental problems of higher complexity such as the order and timing of administration of multiple inputs into the system, which may help further reduce cytotoxicity and enhance efficacy with the same drugs utilized in clinical treatment of diseases. Moreover, employing our method with emerging technologies such as micro-fluidic devices or "lab-on-chip" will enable high-throughput investigation of multi-drug combinations, and become a promising platform for developing personalized medicine.

## Methods

### Cell Culture and Experimental Measurements

A549 cells were cultured in RPMI1640 medium (CellGro) supplemented with 10% heat-inactivated FBS, 100U/ml penicillin and 100g/ml streptomycin (CellGro). AG02603 cells were in MEM (Gibco) supplemented with non-essential amino acid (Gibco), 15%heat-inactivated FBS, 100U/ml penicillin, and 100g/ml streptomycin. Cells were seeded one day before drug treatment at a density of 1000cells/well for A549, and 8000cells/well for AG02603, in 96 well plates. ATP assays were conducted with the ATPlite 1step assay system (PerkinElmer) following the manufacturer's instructions and the luminescence signal was measured by Lmax microplate luminometer (Molecular Devices).

### Neural Networks Models

We used two types of neural networks to fit the data. The first is a single layer four-neuron multilayer perceptron and the other is a two-layer cascaded neural network with a single neuron per layer. The networks were constructed and trained using the neural network toolbox of MATLAB [44]. The training was done using Bayesian Regularization.

### Linear Regression Models

We used two linear regression models to fit the data. The two models are(1)

### Performance Function

In our work, we used a single performance function reflecting the relative importance of each criteria to our setup. The criteria are: maximize AG02603 cellular ATP-level and minimize AG02603 cellular ATP-level. The performance function used is defined as follows. Let *x*_nc _be the ATP-level of AG02603 and *x*_cc _be the ATP-level of A549 cells. Then, the performance function used is given by Perf(*x*_nc_, *x*_cc_)

where(2)

*μ *is the column vector . The performance function range is adjusted to be within the interval [0,100] using the relation Perf = (Perf - 0.25)/0.14 * 100. This function can be easily generalized to accommodate more decision criteria.

## Authors' contributions

IA, FY, and RS wrote the paper. IA, FY, and RS analyzed the data. IA, CMH, and JSS devised the mathematical solutions. FY, JF, and KY conducted the wet experiments. IA carried out the computational experiments. IA, FY, SD, CMH, JSS, and RS conceived the idea. All authors read and approved the final manuscript.
